# Spectrometer‐Less Remote Sensing Image Classification Based on Gate‐Tunable van der Waals Heterostructures

**DOI:** 10.1002/advs.202309781

**Published:** 2024-04-12

**Authors:** Yali Yu, Mianzeng Zhong, Tao Xiong, Jian Yang, Pengwei Hu, Haoran Long, Ziqi Zhou, Kaiyao Xin, Yue‐Yang Liu, Juehan Yang, Jianzhong Qiao, Duanyang Liu, Zhongming Wei

**Affiliations:** ^1^ State Key Laboratory of Superlattices and Microstructures Institute of Semiconductors Chinese Academy of Sciences Beijing 100083 China; ^2^ Center of Materials Science and Optoelectronics Engineering University of Chinese Academy of Sciences Beijing 100049 China; ^3^ Hunan Key Laboratory of Nanophotonics and Devices School of Physics Central South University Changsha Hunan 410083 China; ^4^ School of Automation Science and Electrical Engineering Beihang University Beijing 100191 China; ^5^ School of Instrumentation and Optoelectronic Engineering Beihang University Beijing 100191 China

**Keywords:** alloy engineering, deep learning algorithms, gate‐tunable photodetector, target classification, wide‐spectral

## Abstract

Remote sensing technology, which conventionally employs spectrometers to capture hyperspectral images, allowing for the classification and unmixing based on the reflectance spectrum, has been extensively applied in diverse fields, including environmental monitoring, land resource management, and agriculture. However, miniaturization of remote sensing systems remains a challenge due to the complicated and dispersive optical components of spectrometers. Here, m‐phase GaTe_0.5_Se_0.5_ with wide‐spectral photoresponses (250–1064 nm) and stack it with WSe_2_ are utilizes to construct a two‐dimensional van der Waals heterojunction (2D‐vdWH), enabling the design of a gate‐tunable wide‐spectral photodetector. By utilizing the multi‐photoresponses under varying gate voltages, high accuracy recognition can be achieved aided by deep learning algorithms without the original hyperspectral reflectance data. The proof‐of‐concept device, featuring dozens of tunable gate voltages, achieves an average classification accuracy of 87.00% on 6 prevalent hyperspectral datasets, which is competitive with the accuracy of 250–1000 nm hyperspectral data (88.72%) and far superior to the accuracy of non‐tunable photoresponse (71.17%). Artificially designed gate‐tunable wide‐spectral 2D‐vdWHs GaTe_0.5_Se_0.5_/WSe_2_‐based photodetector present a promising pathway for the development of miniaturized and cost‐effective remote sensing classification technology.

## Graphical abstract

Artificial designed gate‐tunable wide‐spectral 2D‐vdWH GaTe_0.5_Se_0.5_/WSe_2_‐based photodetector, requiring no additional auxiliary components, can achieve an average UV‐Vis‐NIR remote sensing image classification accuracy of 87.00% on 6 prevalent hyperspectral datasets, which is competitive with the accuracy of 250–1000 nm hyperspectral data (88.72%). It presents a promising pathway for the development of miniaturized and cost‐effective remote sensing classification technology.

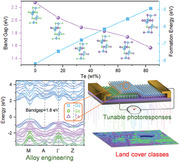

## 1 Introduction

Remote sensing technologies are of great concerns with its non‐intrusiveness, wide‐coverage, and high‐resolution preponderances in numerous fields, including geography, agriculture, land surveying, and environmental monitoring.^[^ ^1^, ^2^, ^3^ ^]^ Hyperspectral imaging is an essential technique in remote sensing, primarily aimed at classifying and recognizing the image using hyperspectral data.^[^ ^4^, ^5^ ^]^ Hyperspectral imaging is usually accomplished by spectrometers which contain bulky optical components (e.g., tunable filter arrays,^[^ ^6^ ^]^ Fourier transform interferometers,^[^ ^7^ ^]^ dispersive grating^[^ ^8^ ^]^).^[^ ^9^ ^]^ Restricted to the complicated and bulky optical components, it is difficult to compact the footprint of the spectrometers to the submicron scale by further optimizing the structure of the optical components.^[^ ^10^, ^11^ ^]^

The utilization of 2D materials to construct novel photoelectronic devices has emerged as a significant strategy for the development of high‐performance and miniaturized devices,^[^ ^12^, ^13^, ^14^ ^]^ benefitting from their distinctive attributes, including their atomic thickness, tunable bandgap, high carrier mobility, and exceptional optoelectronic properties.^[^ ^15^, ^16^, ^17^, ^18^ ^]^ However, the application of photodetectors based on 2D materials in remote sensing encounters certain limitations. Conventional spectrometers possess the capability to identify a broader spectrum and capture the reflectance spectrum with extreme high‐resolution.^[^ ^11^, ^19^, ^20^ ^]^ Reflectance spectral data can introduce a novel dimension into the original 2D image, which enables the classification and identification of surface targets by using the 3D hyperspectral datacubes.^[^ ^10^, ^11^ ^]^ According to literature reports, recent research endeavors have focused on constructing miniature spectrometers using novel 2D semiconductor materials. The underlying principle involves manipulating adjustable parameters to control the spectral response characteristics of the device, including adjusting the composition of the light‐absorbing layer material,^[^ ^21^ ^]^ as well as controlling the gate ^[^ ^10^, ^11^, ^22^ ^]^ or bias^[^ ^23^ ^]^ voltages of the device. Based on the reconstructed series of photoresponse curves, spectral information is obtained, thereby achieving functionality similar to that of commercial spectrometers.

To this end, in order to effectively integrate photodetectors based on 2D materials into the remote sensing systems, with an aim to achieve miniaturization and cost‐effectiveness, it is significant to address two fundamental aspects. First, it becomes imperative to expand the photoresponse range of 2D materials in order to comprehend a broader spectrum of information.^[^ ^24^ ^]^ Second, it is essential to devise a new response data dimension of the photodetector.^[^ ^25^ ^]^ Specifically, the new dimension can be realized through the design and implementation of a gate‐tunable device.^[^ ^10^, ^14^, ^16^, ^26^, ^27^ ^]^ By utilizing multi‐photoresponse of the device along with an artificial neural network to achieve recognition of remote sensing land cover classes without reconstructing spectral information or using conventional hyperspectral data.

Herein, we combine the aforementioned methodologies to construct a wide‐spectral gate‐tunable photodetectors based on the 2D van der Waals heterostructure (*vdWH*) GaTe_0.5_Se_0.5_/WSe_2_. According to the theoretical predictions, the carriers in the alloyed m‐phase *2D* GaTe_0.5_Se_0.5_ nanosheet can achieve multiple transition channels tuned by alloy engineering,^[^ ^28^, ^29^ ^]^ rendering it an ideal candidate for the optical absorption layer in the 2D‐vdWH architecture to realize wide‐spectral photoresponse. Benefitting from the low lattice mismatch, sharp atomic interface, efficient charge and energy transfer, and tailorable band alignment, WSe_2_ is chosen to be stacked with GaTe_0.5_Se_0.5_ to construct the type‐II 2D‐vdWH with tunable electronic and optoelectronic properties. Furthermore, utilizing the multi‐photoreponse data obtained by GaTe_0.5_Se_0.5_/WSe_2_‐based photodetector under varying gate voltages, an artificial neural network is trained for the classification and recognition of remote sensing targets, which can predict the land cover classes. Remarkably, on 6 widely used hyperspectral remote sensing datasets (https://rslab.ut.ac.ir/data), the wide‐spectral gate‐tunable GaTe_0.5_Se_0.5_/WSe_2_‐based device achieves an impressive average prediction accuracy of 87.00%, which is close to the accuracy (88.72%) achieved by commercially spectrometers capturing 250–1000 nm hyperspectral data and much higher than the accuracy (71.17%) achieved by non‐tunable photoresponse. This approach provides significant promise for scaling the remote sensing device footprints down to the micrometer scale and makes noteworthy contributions to the realm of remote sensing image classification.

## 2 Results and Discussion

**Figure 1:**
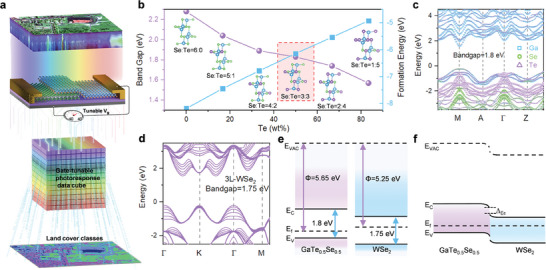
a) Schematic of remote sensing image classification through gate‐tunable wide‐spectral photodetector. b) The energy band gap and formation energy vary with the changing proportion of Te doping in the GaSe. c) The atom‐dependent projected electronic band structure of the GaTe_0.5_Se_0.5_. d) Energy band structure of 3L‐WSe_2_. e,f) Band alignment of the 2D‐vdWH GaTe_0.5_Se_0.5_/WSe_2_ before e) and after f) the contact.

The mechanism for achieving targets classification based on *2D‐vdWH* photodetectors relies on the modulation of multi‐photoresponses through gate voltage control. To improve the accuracy of photodetectors for hyperspectral remote sensing image classification, two crucial factors need consideration: the photoresponse range of the constructed device is wide enough and the photoresponse can be tuned through varying gate voltages. Based on the analysis, gate‐tunable *2D‐vdWH* stacked with semiconductors with wide‐spectral photoresponse properties exhibit significant application potential in this regard.^[^ ^33^ ^]^ **Figure** **1**a illustrates the operational mechanism of target classification in remote sensing image, integrating artificial neural networks (ANN) with multi‐photoresponse data acquired from gate‐tunable wide‐spectral photodetectors. The hyperspectral data in the remote sensing dataset serves as a set of wide‐spectral light illuminating the photodetector. Under the illumination of the wide‐spectral light, the photodetector will output a definite photocurrent value at each pixel of the hyperspectral image. The photocurrent value can be calculated using the formula:

(1) $$I\;=\int_{\lambda_{min}}^{\lambda_{max}}S_{i}\left(\lambda \right)\cdot I_{i}\left(\lambda \right)d\lambda \;$$

in which, if the illumination spectral range is smaller than the photoresponse spectral range of the device, λ_*max*_ and λ_*min*_ represent the maximum and minimum wavelengths of the illumination spectrum, respectively; if the illumination spectrum range exceeds the response spectrum range of the device, λ_*max*_ and λ_*min*_ correspond to the maximum and minimum photoresponse wavelengths of the device, respectively. *S_i_* (λ) denotes the intensity of reflectance spectrum from the remote sensing dataset, and *I_i_* (λ) represents the unit photocurrent generated by the device. A set of photocurrent data corresponding to hyperspectral image pixels can be computed by applying the above formula. By tuning gate voltages of the photodetector, several groups of wide‐spectral photoresponse curves can be obtained, forming a 3D photoresponse data cube. The constructed photoresponse data cube can be trained by using an ANN for land cover classification in remote sensing fields.

Figure 1b demonstrates the effective modulation of bandgap widths and further broadening of the optical absorption range in GaSe through alloying engineering (Figure S1, Supporting Information), by replacing the element Se with the element Te. Additionally, for GaTe, doping with the element Se can effectively reduce the formation energy, making the alloy GaTe_x_Se_1‐x_ more stable. The substitution of Se with Te is attributed to the relatively lower electronegativity of Te, leading to a decrease in electron binding energy.^[^ ^34^ ^]^ Consequently, the electron delocalization tendency becomes stronger upon aggregation with the element Ga to form a lattice structure. Figure 1c depicts the projected energy band structure of the multi‐layered alloy GaTe_0.5_Se_0.5_. Through first‐principles calculations,^[^ ^32^, ^33^ ^]^ it is predicted as a direct bandgap semiconductor with the energy bandgap of 1.8 eV and the atomic contributions of elements Ga, Te, and Se are also shown. The depicted diagram visually demonstrates that, after doping the element Te, the energy levels of the element Te can occupy higher energy states compared to Se. Consequently, electrons are more prone to transition into the conduction band, effectively reducing the bandgap width of the material. This conclusion is further supported through the corroborative density of states (*DOS*) plot corresponding to GaTe_0.5_Se_0.5_, as presented in Figure S2 (Supporting Information). Although the contribution of element Se around the vicinity of the conduction‐band minimum (*CBM*) and valence‐band maximum (*VBM*) is minor, it can still provide additional carrier transition channels upon excitation by photons at specific energies. The transition dipole moment (*TDM*) was utilized to investigate the transition probability of carriers jumping from the valence band to the specific energy levels in the conduction bands, as illustrated in Figure S3 (Supporting Information). A larger *TDM* value signifies a more substantial interaction between the electronic states and the external field, consequently leading to an increased probability of carrier transition. The specific energy levels are denoted by purple lines, while the potential transition channels are depicted by blue arrows. Additionally, there may also be absorption of long‐wavelength light due to defect energy levels. The multiple transition channels in GaTe_0.5_Se_0.5_ broaden the photoresponse range of the photodetector constructed with GaTe_0.5_Se_0.5_ as the optical absorption layer. Notably, in this study, a typical transition metal dichalcogenide compound, WSe_2_, was obtained through the method of mechanical exfoliation, where WSe_2_ nanosheets with appropriate layer numbers were obtained and utilized to construct *2D‐vdWH* with GaTe_0.5_Se_0.5_ nanosheets. The energy band structure of WSe_2_ is illustrated in Figure 1d. The stacked *2D‐vdWH* GaTe_0.5_Se_0.5_/WSe_2_, featuring type‐II band alignment, facilitates distinctive and tunable interlayer and intralayer transition. Band alignments of GaTe_0.5_Se_0.5_/WSe_2_ before and after contact are displayed in Figure 1e and f. The work functions Φ of GaTe_0.5_Se_0.5_ and WSe_2_ nanosheets, determined by ultraviolet photoelectron spectroscopy (UPS),^[^ ^34^ ^]^ are measured to be 5.65 eV and 5.25 eV, respectively (shown in Figure S4, Supporting Information). Considering the theoretically predicted bandgap width and experimentally measured work functions of GaTe_0.5_Se_0.5_ and WSe_2_ nanosheets, Figure 1e illustrates the typical type‐II band alignment in equilibrium state with large band offset. Photoinduced electrons in *CBM* of GaTe_0.5_Se_0.5_ are more easily transferred to the conduction‐band of WSe_2_ benefitting from the positive impact on the large band offset after formation the *2D‐vdWH* GaTe_0.5_Se_0.5_/WSe_2_. An internal built‐in electric field is shown in Figure 1g, which can be effectively tuned by gate voltages to facilitate carrier separation and transport simultaneously. Based on the above analysis, it can be concluded that the photodetector constructed with the *2D‐vdWH* GaTe_0.5_Se_0.5_/WSe_2_ is an ideal choice to achieve land cover classification of hyperspectral remote sensing images.

**Figure 2:**
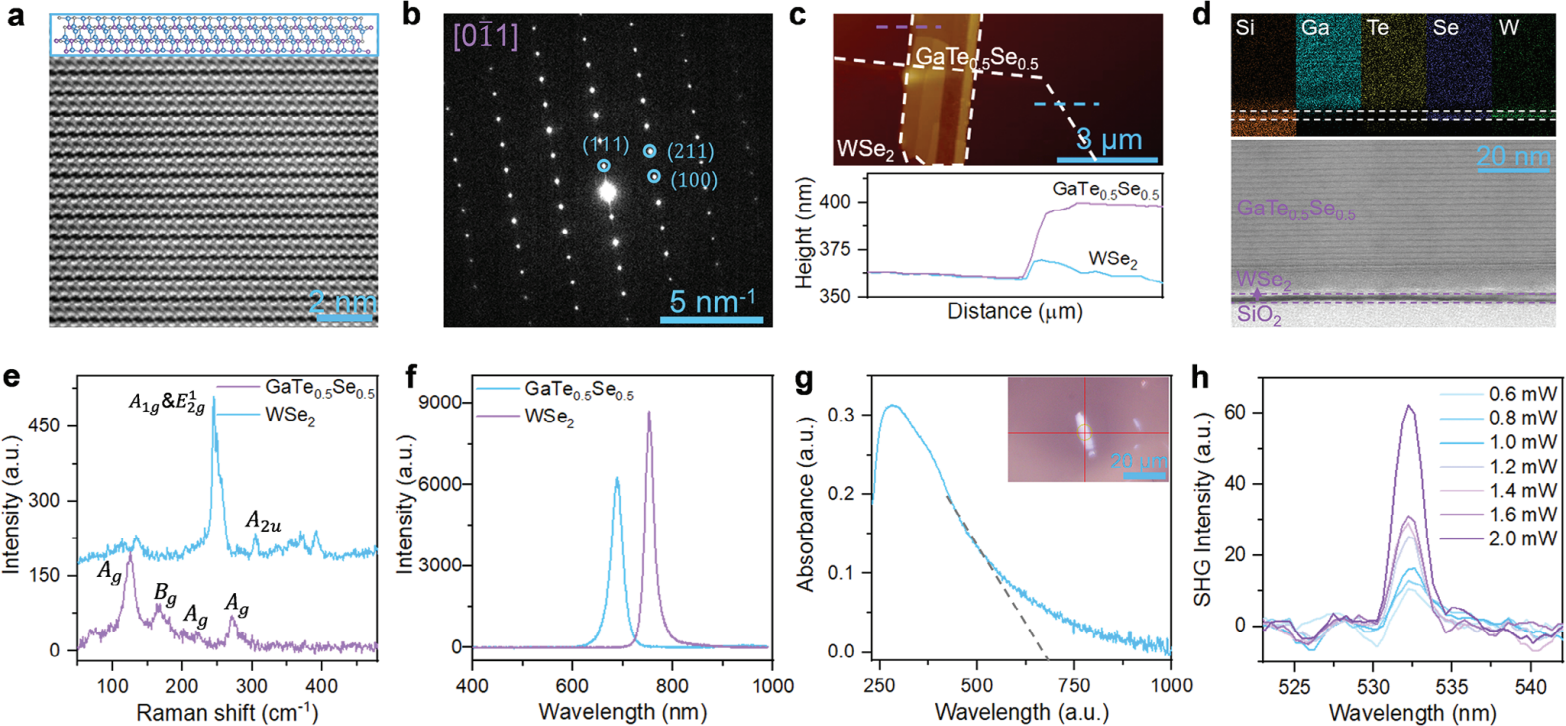
a) HR‐STEM image of GaTe_0.5_Se_0.5_ nanosheet overlapped with a projected atomic model. b) Corresponding SAED image of GaTe_0.5_Se_0.5_ nanosheet. c) Thicknesses of the GaTe_0.5_Se_0.5_ and WSe_2_ nanosheets measured by AFM are ≈40 and 3 nm, respectively. d) Cross‐section TEM image of 2D‐vdWH GaTe_0.5_Se_0.5_/WSe_2_ and corresponding EDS mapping. e) Raman spectra of GaTe_0.5_Se_0.5_ and WSe_2_ nanosheets. f) Measured PL spectra of GaTe_0.5_Se_0.5_ and WSe_2_ nanosheets at room temperature, under an excitation power of 20 µW with a 405 nm laser. g) Measured absorption spectrum of the GaTe_0.5_Se_0.5_ nanosheet. Inset is the corresponding optical microscopy image of the tested GaTe_0.5_Se_0.5_ nanosheets. h) SHG spectra of GaTe_0.5_Se_0.5_ nanosheet with varied power densities under 1064 nm pulsed‐laser excitation.

The projected atomic models of GaTe_0.5_Se_0.5_ in different planes were shown in Figure S5 (Supporting Information). Scanning transmission electron microscopy (*STEM*) was employed to characterize the lattice structure and atomic arrangement of the GaTe_0.5_Se_0.5_ nanosheet primarily. Elemental mapping using energy dispersive X‐ray spectroscopy (*EDS*) was conducted on the low‐resolution *TEM* GaTe_0.5_Se_0.5_ nanosheet (Figure S6a, Supporting Information), revealing a uniform distribution of elements Ga, Te, and Se across the scanned area (Figure S6b–d, Supporting Information). The stoichiometric ratio of Ga, Te, and Se was determined to be 2:1:1 (Figure S6e,f, Supporting Information), which is consistent with the results obtained from X‐ray photoelectron spectroscopy (*XPS*), as shown in Figure S7 (Supporting Information). Selected area electron diffraction (*SAED*) of the GaTe_0.5_Se_0.5_ nanosheet exhibited a distinctive set of regularly arranged diffraction patterns (**Figure** **2**b), with a calculated lattice spacing consistent with [$0\bar{1}1$] direction. Additionally, a high‐resolution *TEM* (*HR‐TEM*) image (Figure 2a) provided detailed information on the lattice spacing and atomic arrangement, confirming that GaTe_0.5_Se_0.5_ belongs to the monoclinic phase. The atomic arrangement of the other orientation was shown in Figure S8 (Supporting Information). Vertical‐structured *2D‐vdWH* were fabricated using a mechanical stacking method, combining multilayered GaTe_0.5_Se_0.5_ nanosheets with few‐layered WSe_2_ nanosheets. The thicknesses of the resulting heterojunction were characterized using atomic force microscope (*AFM*), as shown in Figure 2c. The GaTe_0.5_Se_0.5_ and WSe_2_ nanosheets exhibited thicknesses of ≈40 and 3 nm, respectively. A cross‐section *TEM* image and corresponding *EDS* mapping of *2D‐vdWH* GaTe_0.5_Se_0.5_/WSe_2_ were presented in Figure 2d. The layered structures of GaTe_0.5_Se_0.5_ and WSe_2_ were obviously, while an ≈3 nm thick amorphous layer was detected at the GaTe_0.5_Se_0.5_/WSe_2_ interface. This observation can be attributed to the presence of residual material during the transfer processing, which may negatively impact the collection efficiency and overall device performance.^[^ ^35^ ^]^ Furthermore, the Raman spectrum was employed to identify the phase of the GaTe_0.5_Se_0.5_ nanosheet. Given the limited existing literature on the detailed characterization of GaTe_0.5_Se_0.5_, we present herein preliminary evidence regarding the Raman peak positions. For the multilayer GaTe_0.5_Se_0.5_, four distinct characteristic peaks can be observed at ≈127 ($A_{1g}^{1}$ mode), ≈173, ≈220 ($E_{2g}^{2}$ mode), and ≈278 cm^−1^ ($A_{1g}^{2}$ mode) under 532 nm laser excitation, as depicted in Figure 2e. These observations align with the observed shift trend analyzed in this work.^[^ ^36^ ^]^ The positions of these four Raman characteristic peaks are consistent with the M‐phase GaTe_1‐x_Se_x_ series, further corroborating the findings obtained from the above‐mentioned TEM analysis. Regarding the few‐layered WSe_2_ nanosheet, the out‐of‐plane A_1g_ mode (≈259 cm^−1^) and in‐plane $E_{2g}^{1}$ (≈249 cm^−1^) mode are in excellent agreement with the previously reported findings.^[^ ^37^, ^38^ ^]^ Figure 2f illustrates the photoluminescence (*PL*) spectroscopy of WSe_2_ and GaTe_0.5_Se_0.5_ under 405 nm laser excitation. Notably, exciton resonance peaks can be observed at 690 (GaTe_0.5_Se_0.5_: blue line in Figure 2f) and 752 nm (WSe_2_: purple line in Figure 2f), consistent well with the optical bandgap values *E_g_* = 1240/λ  obtained from the theoretical calculation. To investigate the optical bandgap of the GaTe_0.5_Se_0.5_ nanosheet at room temperature, the absorption spectrum was investigated. As shown in Figure 2g, a wide absorption range of 250 to 1000 nm was observed, featuring a distinct peak at ≈300 nm. Analysis of the absorption spectrum revealed an absorption edge located at ≈690 nm for the GaTe_0.5_Se_0.5_ nanosheet, roughly in agreement with the theoretical predictions. The nonlinear optical responses of the GaTe_0.5_Se_0.5_ nanosheet was investigated in Figure 2h. Under excitation by a 1064 nm femtosecond pulse‐laser, a prominent pick at 532 nm emerges, corresponding to the second harmonic generation (*SHG*) process. Notably, an increase in the incident pulse‐laser power led to a corresponding monotonic increase in the intensity of the *SHG* peak.

**Figure 3:**
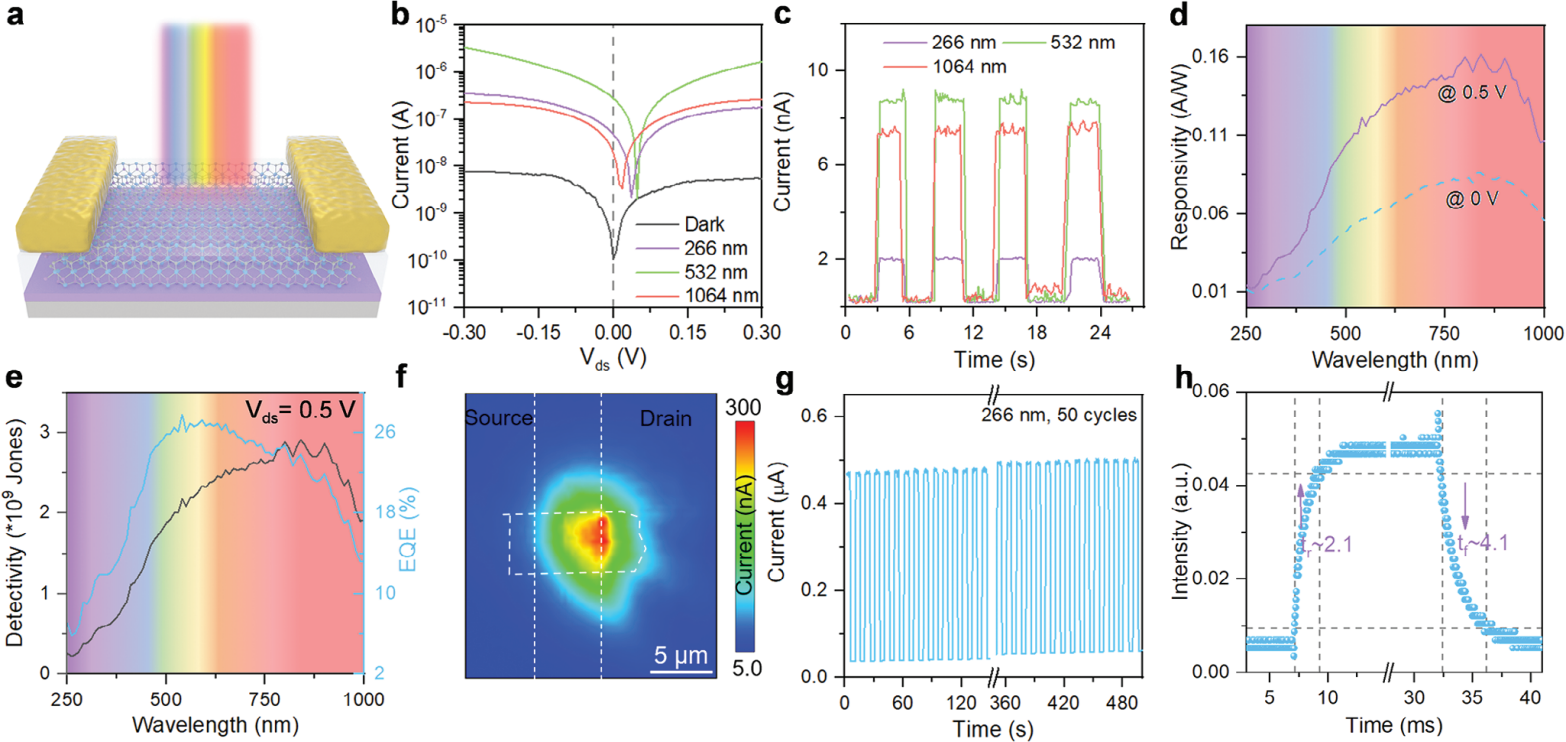
a) Schematic of device structure. b) The I–V curves of the Au/GaTe_0.5_Se_0.5_/Au device under dark condition and illuminations with 266, 532, and 1064 nm lasers (from UVC to NIR bands). c) Photo‐to‐dark current characteristics of the GaTe_0.5_Se_0.5_‐based device at λ = 266, 532, and 1064 nm laser illumination with 0 V bias. d) Spectral photoresponse of the Au/GaTe_0.5_Se_0.5_/Au device under the bias conditions of 0 and 0.5 V. e) Detectivity and EQE of the Au/GaTe_0.5_Se_0.5_/Au device at 0.5 V bias in the spectrum range of 250 to 1000 nm. f) Spatial photocurrent mapping by scanning with the light beam power 1 µW and spot size 1 µm^2^. g) Stability of the GaTe_0.5_Se_0.5_‐based device after more than 50 on‐off cycles at 266 nm laser illumination. The device was measured at the V_ds_ = 0.5 V. h) Time‐resolved photoresponse (rise time: 2.1 ms; fall time: 4.1 ms) of GaTe_0.5_Se_0.5_‐based device at 532 nm laser illumination.

To further investigate the wide‐spectral photoresponse capability of GaTe_0.5_Se_0.5_ nanosheets, the GaTe_0.5_Se_0.5_ was employed as the active photosensitive section to construct the photodetector. The architecture of the GaTe_0.5_Se_0.5_‐based single‐pixel photodetector is depicted in **Figure** **3**a. GaTe_0.5_Se_0.5_ nanosheets were obtained through mechanically exfoliated, with a thickness of ≈40 nm, and then transferred onto the top of the silicon substrate topped with a 300 nm SiO_2_ layer (see Methods for details). Electrodes were further formed at both ends of the GaTe_0.5_Se_0.5_ through photolithography technology. Back‐gated field‐effect transistors (*BG‐FETs*) based on GaTe_0.5_Se_0.5_ were further constructed by assembling GaTe_0.5_Se_0.5_ on the Si/SiO_2_ substrate using SiO_2_ as the gate dielectric. Figure 3b shows the device output I‐V characteristic curves under illumination at different wavelengths ranging from ultraviolet (UV: 266 nm) to near‐infrared (NIR: 1064 nm). Rectifying behavior of the device can be observed in the dark‐state I‐V characteristics. Under a fixed power density of 30 mW cm^−2^, near‐photovoltaic effect was induced, resulting in an open‐circuit voltage (*V_oc_*) of ≈80 mV. The photoresponses of the device in different wavelengths were distinguishable. The output photocurrent of the device under laser illumination of 532 nm (Vis band) surpasses than that under UV and NIR laser illuminations, which was confirmed by the photo‐to‐dark current ratio (*PDCR*) at 0 bias voltage as shown in Figure 3c. To further explore the photoresponse characteristics of the device across the entire UV to NIR wavelength range, spectral responsivity (*R*) under the bias conditions of 0 and 0.5 V were measured within the wavelength range of 250 to 1000 nm, as shown in Figure 3d. Analysis of the result reveals a conspicuous photoresponse within the tested wavelength range, thereby providing further confirmation of the GaTe_0.5_Se_0.5_‐based photodetector's wide‐spectral photoresponse capability. To elucidate two additional important parameters that characterize the performance of the photodetector, namely detectivity (D*) and external quantum efficiency (*EQE*), we conducted preliminary investigations during the experimental process, and the results are presented in Figure 3e. The *EQE* represents the ratio of the number of photo‐generated carriers produced per unit time to the number of incident photons per unit time. What's more, D* effectively measures the photodetector's capability to detect weak light signals and can be calculated using the following formula:

(2) $$D^{*}=R\sqrt{A}/\sqrt{2eI_{dark}}\;$$

where *R* denotes the responsivity of the device, A is the active sensitive area of the device, e is the electronic charge, and I_dark_ is the dark current of the device. Figure 3e displays the D*(λ) of the device within the wavelength range of 250 to 1000 nm. Under a bias condition of 0.5 V, the peak of D* occurs at an incident light wavelength (λ) of 850 nm, and the value is ≈2.9 × 10^9^ Jones. Consequently, we can deduce that the device exhibits a robust capability for detecting weak light signals. The η_*EQE*_ can be calculated using the following the formula:

(3) $$\eta_{EQE}=\;R\times \frac{hc}{e\cdot \lambda }$$

Where h and c are the Planck constant and velocity of light, with values of 4.13 × 10^15^ eV.s and 3 × 10^8^ m ^−1^s, λ is the wavelength of the light source. Based on the calculations from the aforementioned formula (3), η_*EQE*_ of the device is relatively modest, which could be attributed to the low absorption coefficient or the thin thickness of the GaTe_0.5_Se_0.5_ nanosheets.^[^ ^39^ ^]^ More detailed performance parameter values under illumination of different wavelengths are shown in Table S1 (Supporting Information). Figure 3f demonstrates the spatial photocurrent mapping results at λ = 532 nm under the bias conditions of 0 V, further confirming the near‐photovoltaic behavior of the device upon illumination. High stability and fast response speed are two additional important parameters for achieving excellent photodetection applications. In Figure 3g and h, after more than 50 on‐off switching cycles, the output photocurrent and dark current of the device show relatively stable values within a certain range. By adjusting the switching frequency of the incident laser, the response speed of the device can be further explored. The results indicate that the device exhibits a fast photoresponse, with rise and fall times of 2.1 and 4.1 ms, respectively. Through the comprehensive analysis, the findings provide compelling evidence of the immense potential of the GaTe_0.5_Se_0.5_‐based photodetector in the realm of wide‐spectral photodetection. Moreover, employing band engineering methodologies, including the formation of *2D‐vdWH* by strategically stacking GaTe_0.5_Se_0.5_ with other suitable *2D* materials, opens up a realm of possibilities for the development of highly versatile photodetection applications.

**Figure 4:**
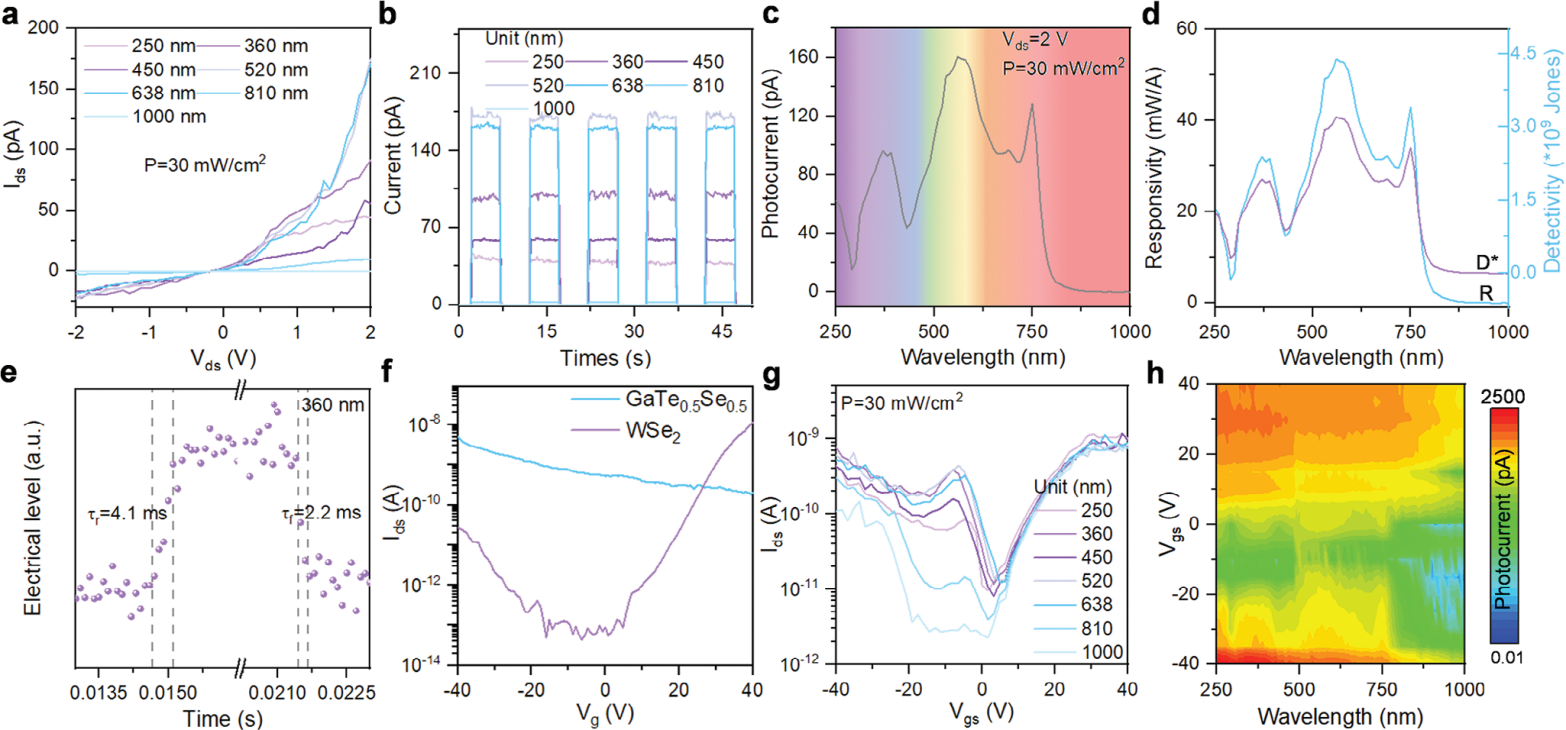
a) The I–V curves of the device under illumination with varying‐wavelength lasers (from UVC to NIR bands). b) Time‐resolved on‐off switching photoresponse under the same conditions as in a). c) Spectral photoresponse of the device under a bias condition of 2 V. d) R and D* of the device at a bias of 2 V bias within the spectral range of 250 to 1000 nm. e) Derived from λ = 360 nm laser illumination, 10–90% photocurrent rise and fall times demonstrate approximately 4.1 and 2.2 ms, respectively. f) Transfer characteristics of the back‐gate‐regulated GaTe_0.5_Se_0.5_ and WSe_2_ under dark conditions. g) Transfer characteristics of the back‐gate‐regulated 2D‐vdWH GaTe_0.5_Se_0.5_/WSe_2_ under light illumination at different wavelengths, with a fixed power density of 30 mW/cm^2^. h) A color contour plot of the spectral photoresponse under varying gate voltage.

The performance of photodetectors based on the *2D‐vdWH* GaTe_0.5_Se_0.5_/WSe_2_ was evaluated under varying gate voltages and laser irradiation in the spectral range of 250 to 1000 nm. The I‐V characteristic curves of the devices were measured at room temperature using the same light power density of 30 mW/cm^2^, as depicted in **Figure** **4**a. The results demonstrated a significant reduction in the dark current of the devices to the pA level after the formation of the *2D‐vdWH*, exhibiting typical rectification characteristics. Statistical analysis of the photocurrent and dark current of six typical devices showed that the devices have good stability within a certain range, as shown in Figure S9 (Supporting Information). Under a gate voltage (*V_gs_*) of 0 V and a source‐drain voltage (*V_ds_*) of 2 V, the devices exhibited maximum output current (*I_ds_*) when the incident laser wavelength was 638 nm. Conversely, at an incident laser wavelength of 1000 nm, the output *I_ds_* closely resembled the dark current (*I_dark_*), indicating minimal discernible photoresponse. Figure 4b illustrated the on‐off switching characteristics of the photodetector. Under the identical conditions as in Figure 4a, time‐resolved on/off photoresponses were performed on the photodetector with laser illumination at different wavelengths for 5 consecutive cycles. The results demonstrated excellent stability of the devices based on *2D‐vdWH* GaTe_0.5_Se_0.5_/WSe_2_, consistent with the conclusions drawn from Figure 4a. The *PDCR* values of the devices were ≈1 × 10^4^ at the incident laser wavelengths of λ = 532 and 638 nm. By adjusting the sampling frequency of the testing equipment, further exploration of the response speed of the photodetectors can be conducted. Under 360 nm laser illumination, the devices exhibited temporal photoresponse with rise and fall times of 4.1 and 2.2 ms, respectively. Comparatively, the *2D‐vdWH* GaTe_0.5_Se_0.5_/WSe_2_‐based photodetectors displayed a photoresponse speed in the millisecond range, similar to the GaTe_0.5_Se_0.5_ counterparts, thus indicating minimal impact on device performance from the amorphous layer at the GaTe_0.5_Se_0.5_/WSe_2_ interface. Additionally, the spectral responsivity was further investigated to validate the photodetection characteristics of the devices in the UV to NIR range (250 to 1000 nm) under 2 V source‐drain bias conditions and 0 V gate voltage, as shown in Figure 4d. More detailed performance parameter values under illumination of different wavelengths are shown in Table S2 (Supporting Information). Subsequently, the transfer characteristics curves of three types of photodetectors, each utilizing GaTe_0.5_Se_0.5_, WSe_2_, and GaTe_0.5_Se_0.5_/WSe_2_ as the active layer, were measured by applying a gate voltage ranging from −40 to 40 V while maintaining a fixed source‐drain voltage of 2 V. The results shown in Figure 4f,g indicate that the photodetector based on GaTe_0.5_Se_0.5_ exhibits a slight p‐type transfer characteristic, whereas the photodetector based on WSe_2_ demonstrates bipolar behavior. In the case of the GaTe_0.5_Se_0.5_/WSe_2_‐based photodetector, a “reverse‐bipolar” behavior was observed under illumination conditions ranging from 250 nm to 810 nm. Additionally, Figure 4g reveals distinct wavelength‐dependent transfer characteristics curves, clearly illustrating the dependence of the output I_ds_ of the device based on *2D‐vdWH* GaTe_0.5_Se_0.5_/WSe_2_ on the V_gs_. To comprehensively explore the wide‐spectral gate‐tunable properties, the incident light was systematically scanned with a precision of 10 nm within the wavelength range of 250 to 1000 nm. Concurrently, the gate voltage of the device was precisely varied in increments of 5 V, spanning from −40 to 40 V. Notably, a comprehensive analysis of the collected multiple output photocurrent values enabled the generation of a comprehensive *2D* contour plot, as shown in Figure 4h. Spectral photoresponse current within the wavelength range of 250 to 1000 nm under varying gate voltages in linear and exponential coordinates were shown in Figure S10 (Supporting Information). The intricate contour plot elucidates the remarkable wide‐spectral gate‐tunable characteristics exhibited by the *2D‐vdWH* GaTe_0.5_Se_0.5_/WSe_2_‐based photodetector. This distinct property enables the integration of the photodetector into artificial neural networks for the purpose of land cover classification and identification of remote sensing image, thereby showcasing its significant utility in such applications.

**Figure 5:**
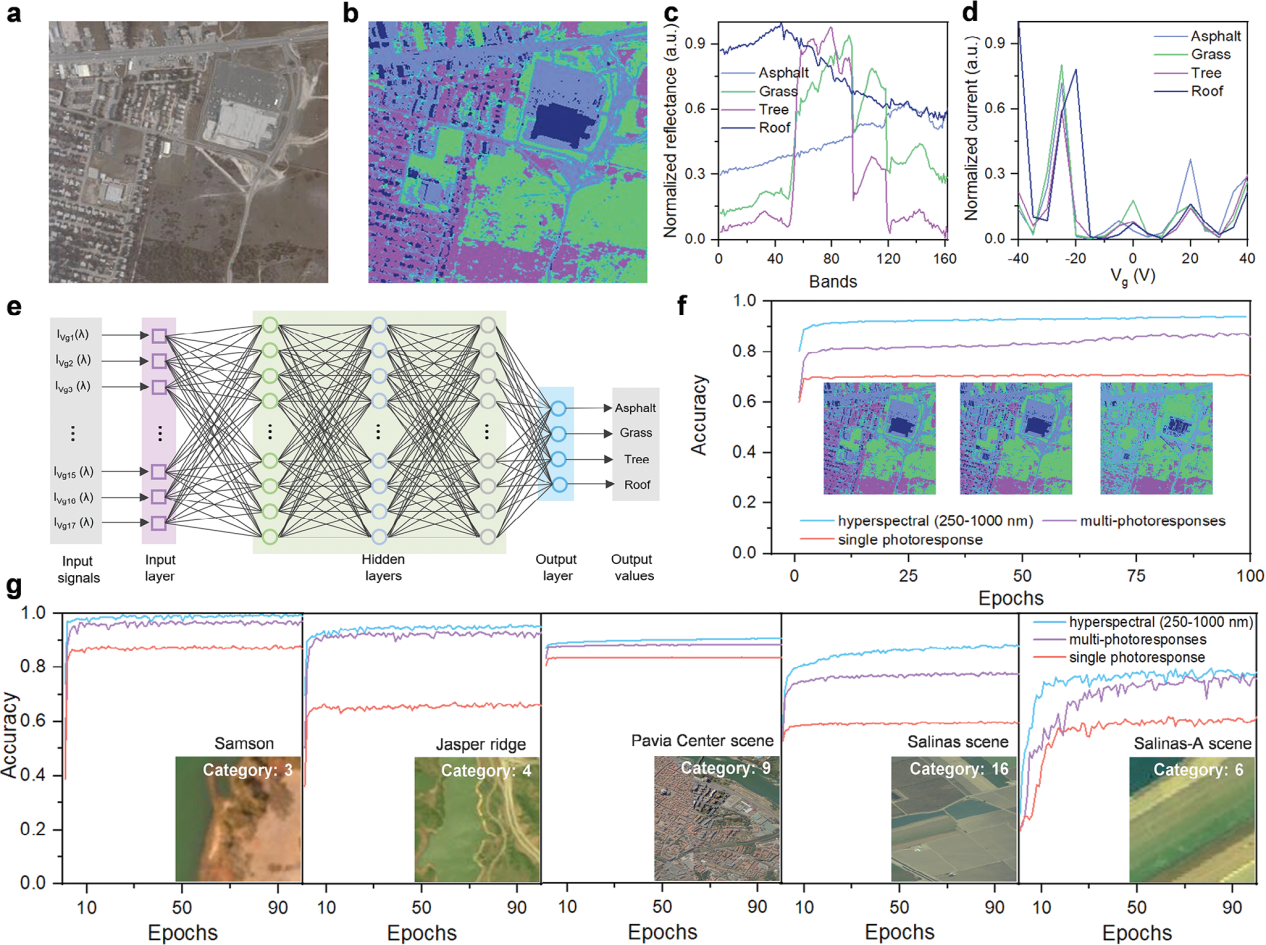
a) The RGB image of the original Urban dataset(https://rslab.ut.ac.ir/data). b) The ground truths of the Urban dataset, consisting of four land cover classes: asphalt, grass, tree, and roof. c) The reflectance spectra of the four land cover classes in the Urban dataset. d) The gate‐tunable multi‐photoresponses to the reflected light of the four land cover classes at different gate voltages from −40 to 40 V. e) Schematic diagram of the structure of the artificial neural network. f) Classification results predicted by the neural network trained with bands information within the photodetector's response range (250–1000 nm) of the hyperspectral datacube, gate‐tunable multi‐photoresponse data of the photodetector, and non‐tunable single photoresponse data of the photodetector at different gate voltages. g) Classification accuracy of different hyperspectral remote sensing image datasets is achieved by using bands information within the photodetector's response range (250 to 1000 nm) of the hyperspectral datacube, gate‐tunable multi‐photoresponse data of the photodetector at different gate voltages, and non‐tunable single photoresponse data of the photodetector as input signals (https://rslab.ut.ac.ir/data).

To demonstrate the application potential of gate‐tunable GaTe_0.5_Se_0.5_/WSe_2_ *2D‐vdWH*‐based photodetectors, an artificial neural network is trained using the gate‐tunable multi‐photoresponse data to solve the remote sensing image classification problem as a proof‐of‐concept, the AI classification flow diagram is shown in Figure S11 (Supporting Information). Building upon the wide‐spectral and gate‐tunable photoresponse and rapid photoresponse speed exhibited by the device, combined with the assured stability of its performance, the methodology for remote sensing image classification using the obtained multi‐photoresponse datacube from the photodetector at various gate voltages is presented in **Figure** **5**, alongside a comparison with results based on hyperspectral datacube and non‐tunable single photoresponse. Figure 5a to f illustrate the complete process of remote sensing image classification using the Urban hyperspectral dataset as a representative example, accompanied by detailed visualizations of the remote sensing data and neural network training process. The RGB image of the Urban dataset is depicted in Figure 5a, comprising a total of 307 × 307 pixels, with each pixel corresponding to a 2 × 2 m^2^ area. Figure 5b displays the ground truth labels of the Urban dataset, consisting of four land cover classes: asphalt, grass, tree, and roof. In Figure 5c, the reflectance spectra of the four land cover classes within the Urban hyperspectral dataset are presented. The original dataset consists of 210 bands ranging from 400 to 2500 nm, resulting in a spectral resolution of 10 nm. Notably, 48 bands were excluded from the dataset due to the presence of dense water vapor and atmospheric effects (https://rslab.ut.ac.ir/data). Figure 5d demonstrates the photoresponse of the photodetector to the reflected light from the four land cover classes at various gate voltages, which can be calculated by the formula: $I\;=\int_{\lambda_{min}}^{\lambda_{max}}S_{i}\left(\lambda \right)\cdot I_{i}\left(\lambda \right)d\lambda$. In which, λ_*min*_ represent the minimum wavelength of the illumination spectrum and λ_*max*_ correspond to the maximum photoresponse wavelength of the device. *S_i_* (λ) denotes the intensity of reflectance spectrum from the remote sensing dataset shown in Figure 5c, and *I_i_* (λ) represents the unit photocurrent generated by the device. As a demonstration, the photoresponse spectra are measured at 17 gate voltages ranging from −40 to 40 V, with a 5 V increment. The results reveal discernible variations in the photoresponses of different objects under different gate voltages. By exploiting the prominent differences in the photoresponse variations among objects under distinct gate voltages, we can ascertain the class membership of objects based on their respective photoresponse trends at different gate voltages. To automate the classification task based on the normalized photocurrent values at various gate voltages, an artificial neural network is devised and shown in Figure 5e, which is comprised of three hidden layers, each housing 1024 neurons. The input signal consists of the photocurrents of a single pixel at 17 distinct gate voltages, derived from the remote sensing dataset. The output values of the network are predicted land cover classes. To conduct comparative experiments, equivalently structured neural networks are trained respectively by using 400–1000 nm hyperspectral data and non‐tunable single photoresponse data. The Urban dataset encompasses 94249 pixels, allocated 80% for training and 20% for validation. The training results with different input signals from the same‐structure neural network are shown in Figure 5f. In addition to training with photoresponse data at various gate voltages, experiments are also conducted by employing complete hyperspectral data and hyperspectral data within the photoresponse wavelength range (400 to 1000 nm). After 100 training epochs, the neural network trained with 400–1000 nm hyperspectral data achieves a classification accuracy of 93.54%, whereas the network trained with non‐tunable single photoresponse data achieves a classification accuracy of 70.80%. Notably, the neural network trained with gate‐tunable multi‐photoresponse data at different gate voltages attains a classification accuracy of 87.40%. The inferred images of the entire remote sensing dataset by the neural networks trained with the three data types are further presented. The images inferred using hyperspectral data and gate‐tunable multi‐photoresponse data exhibit remarkable concordance with the ground truth images displayed in Figure 5b while the image inferred using non‐tunable single photoresponse data exhibits relatively large difference.

Figure 5g exhibits the training curves on other 5 commonly employed remote sensing datasets, namely Samson, Jasper Ridge, Pavia Center, Salinas, and Salinas‐A. After 100 training epochs, the neural network trained with gate‐tunable multi‐photoresponse data at various gate voltages achieved classification accuracies of 96.37%, 93.01%, 88.75%, 78.83%, and 77.61%, respectively. Across the aforementioned 6 remote sensing datasets, the utilization of gate‐tunable multi‐photoresponse data from GaTe_0.5_Se_0.5_/WSe_2_ *2D‐vdWH*‐based photodetectors for training neural networks of identical architecture yielded an average classification accuracy of 87.00%, which represents a 1.72% reduction compared to training with 250–1000 nm hyperspectral data, which amounted to 88.72%. Notably, considering the limited multi‐photoresponse of only 17 gate voltages in the demonstration experiment, the training results of the neural network indicate that the information contained in the photoresponse data at these 17 gate voltages rivals that contained within the hundreds of spectral bands of reflectance intensity in hyperspectral data. In addition, the average classification accuracy of the neural networks training with non‐tunable single photoresponse data is only 71.17%, which illustrates that the gate‐tunable photodetector is much more effective in the remote sensing task compared to non‐tunable photodetector. The land cover classes images of 6 prevalent hyperspectral remote sensing datasets predicted by neural networks training with the hyperspectral data (250‐1000 nm), gate‐tunable multi‐photoresponses, and non‐tunable single photoresponse are shown in Table S3 (Supporting Information). Through the demonstration experiment, the feasibility of deploying gate‐tunable photodetectors as an alternative to spectrometers in remote sensing applications was substantiated. To further augment classification accuracy, future applications can explore finer‐grained gate voltage control and the design of photodetectors with broader spectral photoresponse ranges.

## 3 Conclusion

In summary, we have constructed a wide‐spectral gate‐tunable photodetector based on *2D‐vdWH* GaTe_0.5_Se_0.5_/WSe_2_ that enables UV‐Vis‐NIR remote sensing image classification. The GaTe_0.5_Se_0.5_/WSe_2_‐based photodetector demonstrates wide‐spectral photoresponses (250 to 1000 nm) through the artificially designed m‐phase alloy GaTe_0.5_Se_0.5_ nanosheet with multi‐channel transitions as well as the typically type‐II band alignment of the heterojunction. The device exhibits gate‐tunable characteristic apparently and corresponding photoresponses are obviously distinct under varying gate voltages. This design does not have any integrated dispersive and bulky optical components or any other array structures, the footprint can be effectively scale down to the micron order. By utilizing the gate‐tunable multi‐photoresponses of the GaTe_0.5_Se_0.5_/WSe_2_‐based photodetector built upon the premise of stable performance, an artificial neural network has been trained with the multi‐photoresponse datacube of 6 prevalent remote sensing datasets to achieve a classification accuracy of 87.00%. In comparison to previous methods, this work has promising prospects for further developing the functional and miniaturized electronic devices that can adapt to numerous complicated remote sensing application scenarios without previous spectrometers.

## 4 Experimental Section

### Crystal Growth

The GaTe_0.5_Se_0.5_ crystal was sintered through the chemical vapor transport (CVT) method. The precursors adopted were commercially available liquid gallium (99.999%, Alpha), tellurium powder (99.99%, Alpha), selenium powder (99.99%, Alpha), and iodine (99%, Alpha). The stoichiometric ratio of mentioned powders was 2:1:1. Iodine was used as the transport agent in the growth process. All of precursors were vacuum‐sealed in the quartz tube (10^−3 ^Pa). The quartz tube was then placed in the dual‐zone tube furnace. The reaction source region's temperature was set at 1200 °C, whereas the reaction deposition region's temperature was set at 1000 °C. The furnace chambers were kept at the above‐mentioned temperature for 10000 minutes. Following the growing process, the temperature of the tube furnace was cooled to 200 °C at a rate of 10 °C hour^−1^ and then naturally cooled to room temperature. Eventually, high‐qualitied GaTe_0.5_Se_0.5_ bulk crystal could be obtained.

### Characterization Methods

Angle‐resolved *Raman spectrum*. Angle‐resolved Raman spectrum was performed by Renishaw inVia Reflex Raman Microscope and Spectrometer. The excitation light wavelength was 532 nm. In the angle‐resolved measurement, the polarization direction of the analyzer was parallel (parallel mode) or vertical (cross mode) to the polarized excitation light. Angle‐resolved absorption spectrum. The absorption spectrum was performed by MStarter ABS DUV‐NIR Microscopic Absorption Spectroscopy System. The differential transmission method was used to characterize by comparing the transmittance between the sample and the back substrate. Photoluminescence Spectroscopy. PL spectroscopy was carried out in the Mstarter 100 Microspectral Scanning Test System, and the excitation laser wavelength was 405 nm. Atomic force microscopy. AFM measurements were carried out in the Bruker Dimension Icon scanning probe microscope. The thicknesses of the WSe_2_, GaTe_0.5_Se_0.5_ and WSe_2_/GaTe_0.5_Se_0.5_ vdW heterostructure were characterized in lift mode by MESP probes. X‐ray photoelectron spectroscopy. XPS was carried out in Thermo escalab 250Xi. The amount of each element in the GaTe_0.5_Se_0.5_ single crystal can be quantified. Transmission Electron Microscope. The HR‐TEM, SAED, and element distribution were characterized through it. The cross‐sectional crystallographic sample was obtained by using the focused‐ion‐beam (FIB) technology, which was carried out in FEI Titan cubed Themis G2 300 STEM with a spherical aberration corrector.

### Device Fabrication

GaTe_0.5_Se_0.5_‐based photodetectors were fabricated by starting from mechanically exfoliating GaTe_0.5_Se_0.5_ nanosheets on top of the silicon substrate topped with a 300 nm SiO_2_ layer. Back‐gated field‐effect transistor (BG‐FET) based on 2D‐vdWH GaTe_0.5_Se_0.5_/WSe_2_ were fabricated by assembling WSe_2_ and GaTe_0.5_Se_0.5_ flake by flake on the Si/SiO_2_ substrate. Dry‐release transfer method was adopted using polydimethylsiloxane membranes for 2D‐vdWH GaTe_0.5_Se_0.5_/WSe_2_. The WSe_2_ nanosheets were mechanically exfoliated from the bulk single crystal (Shanghai Onway Technology Co., Ltd). Channel‐masking method was adopted to define the channel of the devices. Then, Au was evaporated with a thickness of 40 nm to form the electrodes through thermal evaporation technology.

### Electrical and Optoelectrical Measurements

The electronic and optoelectronic measurements were conducted using an Agilent Technologies B1500A semiconductor device analyzer combined with the homebuilt optoelectronic measurement platform. The response time of the device were acquired from a AOM Holy light modulator (Gooch&housego 3080–125) and oscilloscope (Rigol MSO5102) with a homebuilt optoelectronic measurement system. The spatial photocurrent mapping was performed by Mstarter 200 High Precision Photocurrent Scanning Test Microscope. All measurements were done at room temperature and repeated more than once to confirm the reproducibility.

### Computational Details

The electronic band structure of GaSe_1‐x_Te_x_ was calculated by HSE06 functional under the highly symmetric path of L→M → A →Γ →Z →V. First‐principles calculations were carried out based on density functional theory (DFT) using the Vienna ab initio simulation package (VASP). The projector augmented wave (PAW) method was used to describe the core electrons. The generalized gradient approximation (GGA) with Perdew‐Burke‐Ernzerhof (PBE) functional was adopted to treat the exchange‐correlation potential, and the HSE06 (Heyd‐Scuseria‐Ernzerhof) hybrid functional was used to obtain more accurate electronic band structures. A kinetic energy cutoff of 520 eV and a Monkhorst‐Pack k–mesh of 9×9×3 sampling in the full Brillouin zone was used in the calculations. The structures were optimized until all residual forces on each atom were smaller than 0.01 eV Å^−1^. The simulations were performed at 0 K.

## Conflict of Interest

The authors declare no conflict of interest.

## Supporting information

Supporting Information

## Data Availability

Research data are not shared.
