# Supplementary material for: Spectrometer‐Less Remote Sensing Image Classification Based on Gate‐Tunable van der Waals Heterostructures
Source: Adv Sci (Weinh). 2024 Apr 12;11(24):2309781. doi: 10.1002/advs.202309781 (PMC11200008; doi:10.1002/advs.202309781)
Supplement: Supplementary file 1 — Supporting Information [file ADVS-11-2309781-s001.pdf]

ADVANCED SCIENCE
Open Access

Supporting Information

for *Adv. Sci.*, DOI 10.1002/advs.202309781

Spectrometer-Less Remote Sensing Image Classification Based on Gate-Tunable van der Waals Heterostructures

Yali Yu, Mianzeng Zhong, Tao Xiong, Jian Yang, Pengwei Hu, Haoran Long, Ziqi Zhou, Kaiyao Xin, Yue-Yang Liu, Juehan Yang, Jianzhong Qiao*, Duanyang Liu* and Zhongming Wei**

Supporting Information for:

Spectrometer-less remote sensing image classification based on gate-tunable van der Waals heterostructures

Yali Yu, Mianzeng Zhong, Tao Xiong, Jian Yang, Pengwei Hu, Haoran Long, Ziqi Zhou, Kaiyao Xin, Yue-Yang Liu, Juehan Yang, Jianzhong Qiao*, Duanyang Liu*, Zhongming Wei**

Y. Yu, T. Xiong, H. Long, Z. Zhou, K. Xin, Prof. Y-Y. Liu, Dr. J. Yang, Dr. D. Liu, Prof. Z. Wei
State Key Laboratory of Superlattices and Microstructures, Institute of Semiconductors, Chinese Academy of Sciences, Beijing 100083, China
Email: liudy@semi.ac.cn; zmwei@semi.ac.cn

Y. Yu, T. Xiong, H. Long, K. Xin, Prof. Z. Wei
Center of Materials Science and Optoelectronics Engineering, University of Chinese Academy of Sciences, Beijing 100049, China

Prof. M. Zhong
Hunan Key Laboratory of Nanophotonics and Devices, School of Physics, Central South University, Changsha 410083, Hunan, China

Dr. J. Yang, Prof. J. Qiao
School of Automation Science and Electrical Engineering, Beihang University, Beijing 100191, China
E-mail: jzqiao@buaa.edu.cn

Dr. P. Hu
School of Instrumentation and Optoelectronic Engineering, Beihang University, Beijing 100191, China
E-mail: hupengwei@buaa.edu.cn

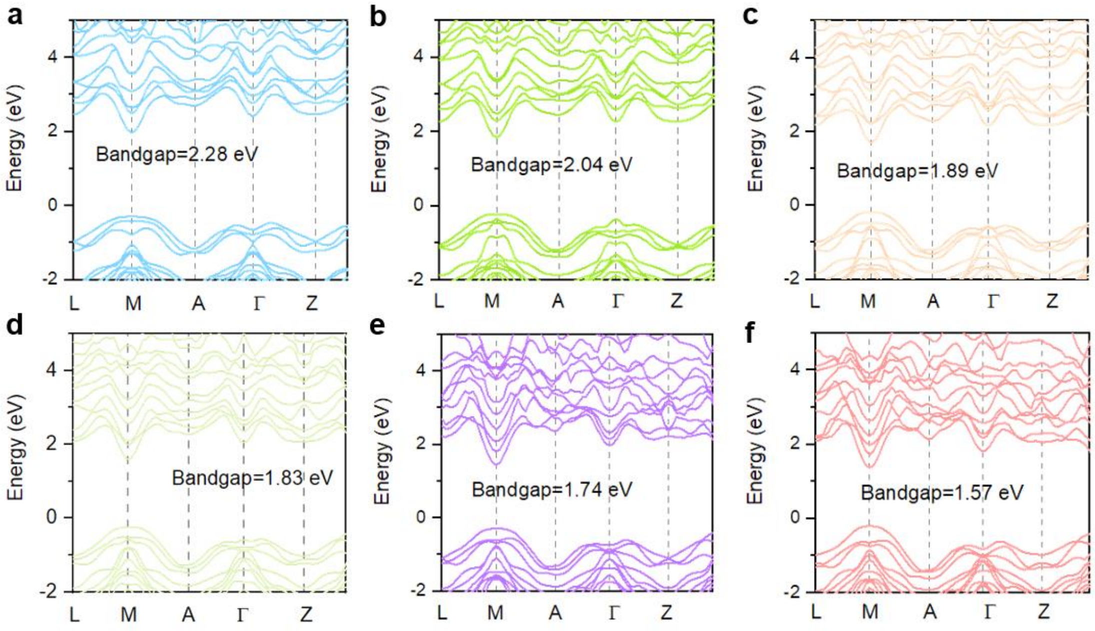

Figure S1. The theoretical calculated energy band structure of different ratios between Se and Te. (a) Se:Te =6:0. (b) Se:Te =5:1. (c) Se:Te =4:2. (d) Se:Te =3:3. (e) Se:Te =2:4. (f) Se:Te =1:5.

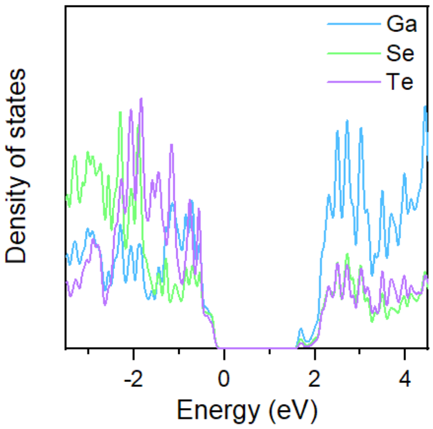

Figure S2. The projected density of charge states of the bulk $GaTe_{0.5}Se_{0.5}$ of the elements Ga, Te, and Se.

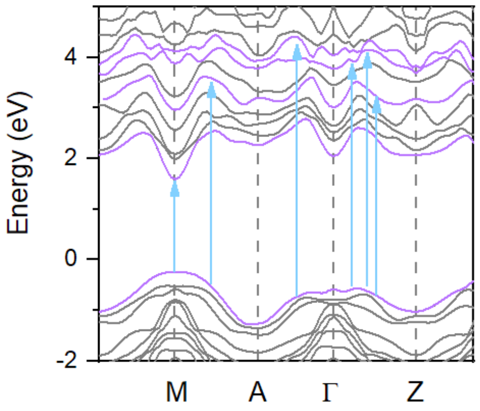

Figure S3. Energy band structure of $GaTe_{0.5}Se_{0.5}$ and specific carriers transition channels predicted by the TDM.

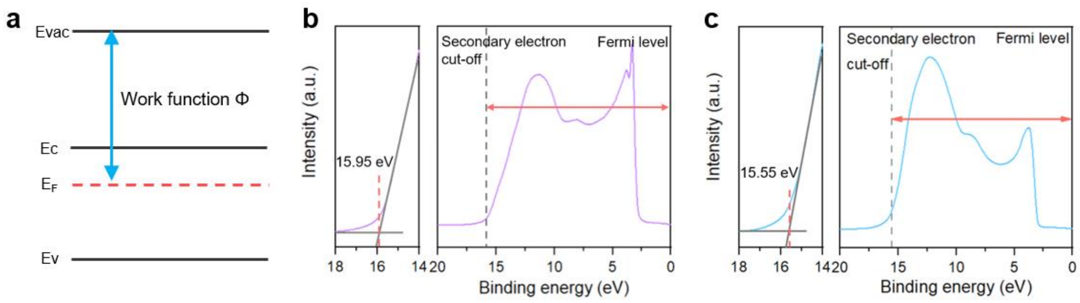

Figure S4. (a) Schematic illustration of the energy diagram. (b) The Micro zone ultraviolet photo-electron spectroscopy (UPS) spectrum of 3L-WSe_2 nanosheets. (c) The UPS spectrum of multi-layered $GaTe_{0.5}Se_{0.5}$ nanosheets. The left panel shows the magnified view of the high binding energy region and gray lines in panel represent the linear fits.

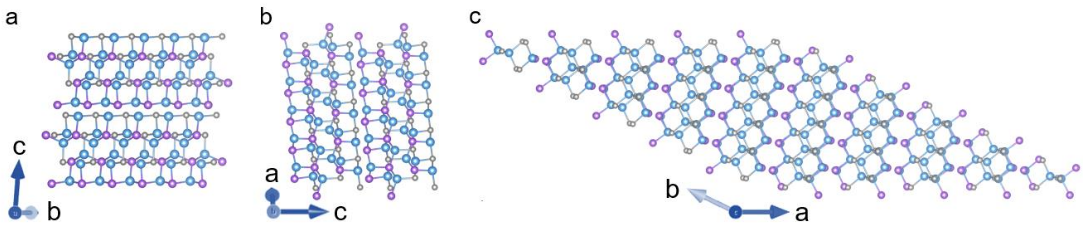

Figure S5. The projected atomic models of $GaTe_{0.5}Se_{0.5}$ in different planes. (a) b-c plane. (b) a-c planes. (c) a-b plane.

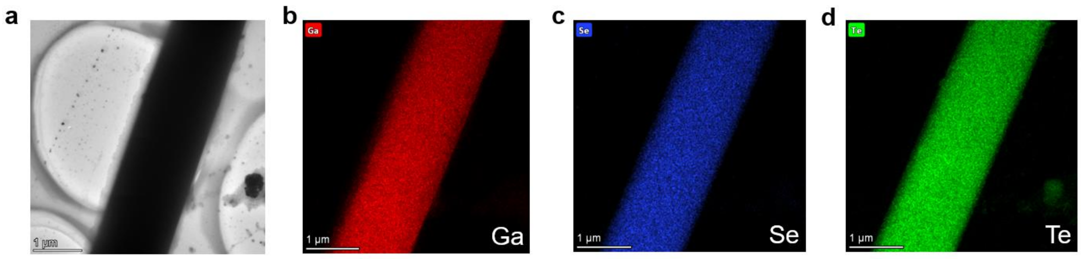

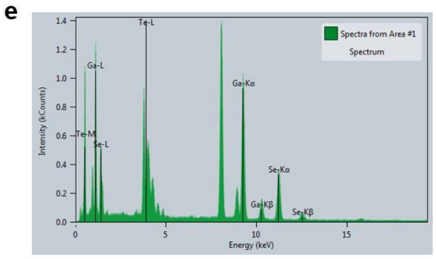

f

Element	Family	Atomic fraction
Ga	K	50%
Se	K	24%
Te	L	26%

Figure S6. Morphology and component characterizations. (a) Low-magnification TEM image of $GaTe_{0.5}Se_{0.5}$ nanosheet. (b-d) Energy-dispersive spectroscopy (EDS) element mapping of Ga, Te, and Se elements. (e) EDS of the $GaTe_{0.5}Se_{0.5}$ nanosheet. (f) atomic fraction of elements Ga, Te, and Se in $GaTe_{0.5}Se_{0.5}$ nanosheet.

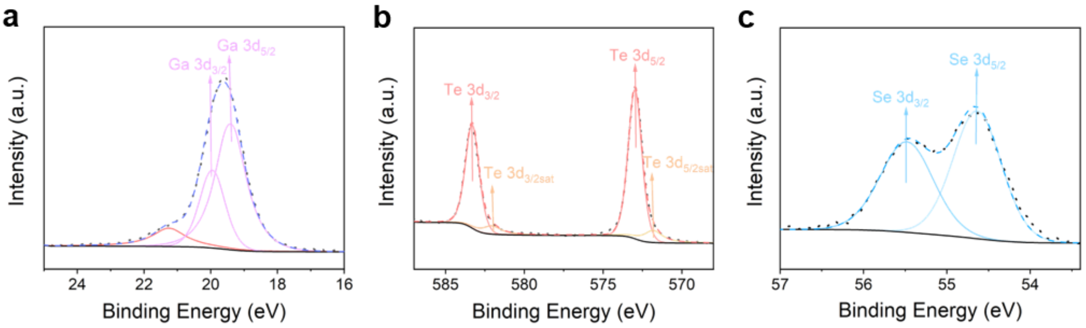

Figure S7. (a) High-resolution spectrum of Ga3d core level. (b) High-resolution spectrum of Te3d core level. (c) High-resolution spectrum of Se3d core level.

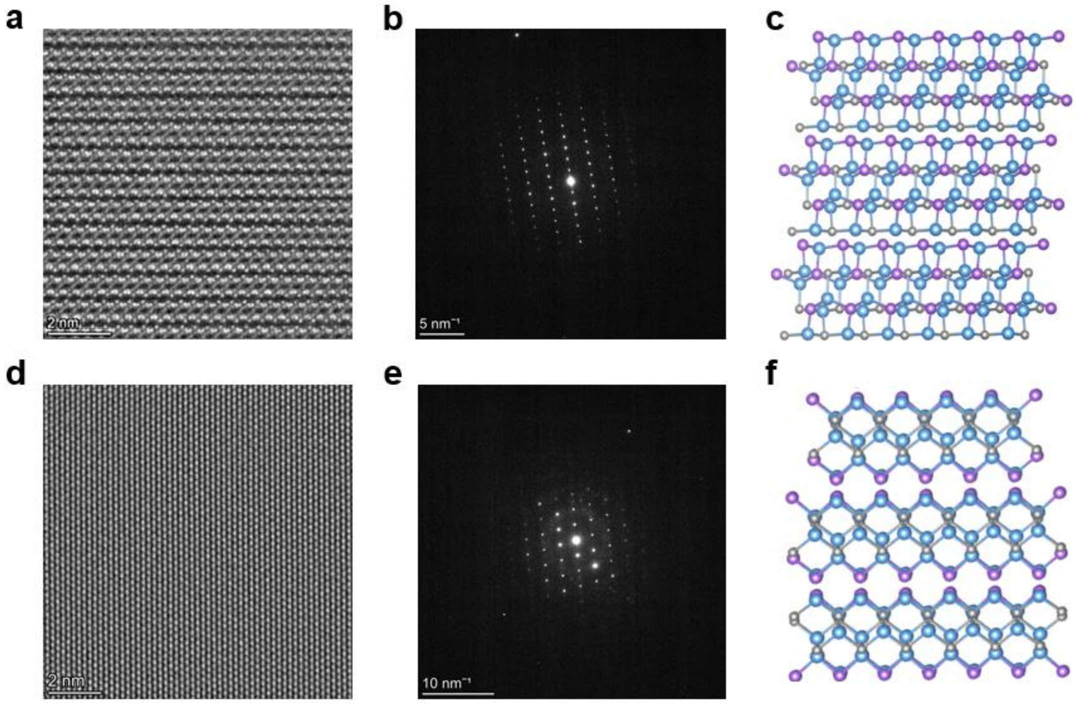

Figure S8. STEM characterization of $GaTe_{0.5}Se_{0.5}$ nanosheets. (a-c) High-resolution cross-sectional STEM image of $GaTe_{0.5}Se_{0.5}$. (a) SAED pattern (b) and corresponding atomic model (c) of $GaTe_{0.5}Se_{0.5}$. (d-f) High-resolution STEM image (d), SAED pattern (e), and corresponding atomic model (f) of $GaTe_{0.5}Se_{0.5}$.

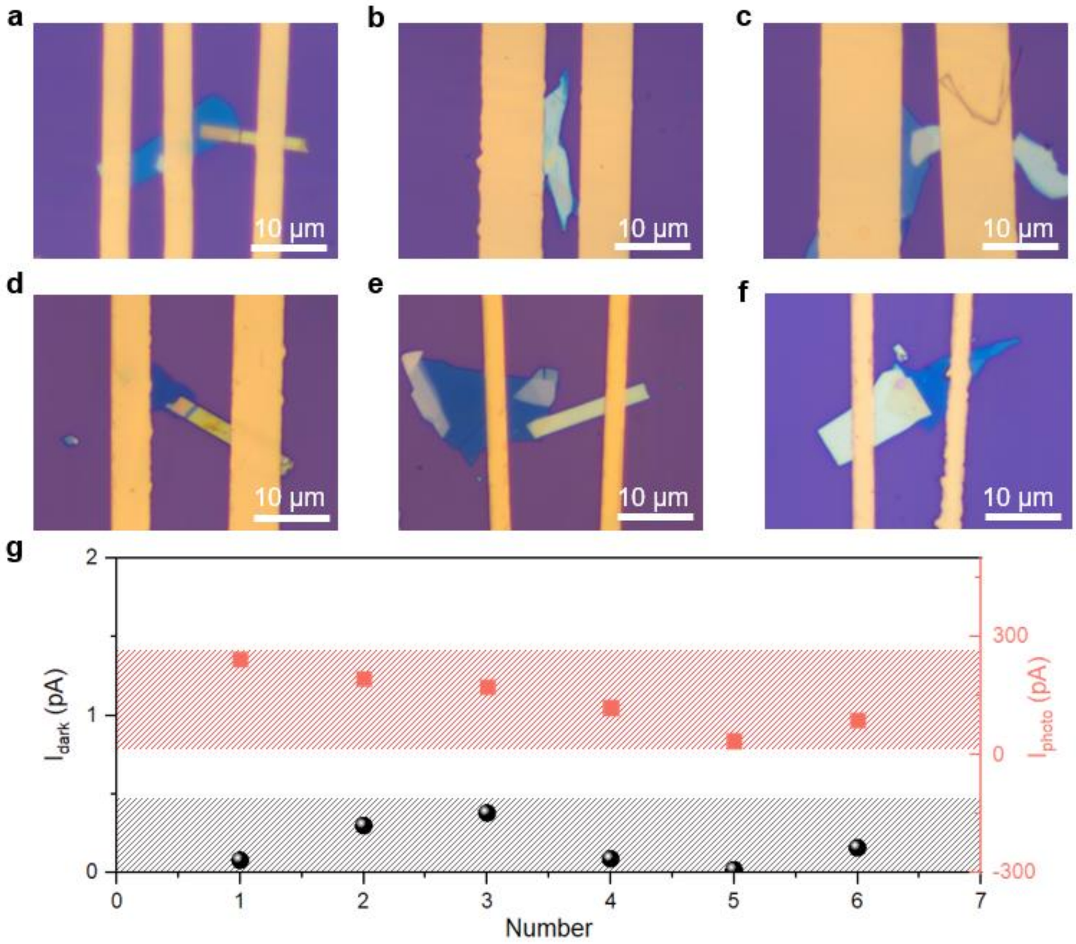

Figure S9. Performance of 6 typical photodetectors based on 2D-vdWH $GaTe_{0.5}Se_{0.5}/WSe_2$. (a-f) Microscope image of 6 $GaTe_{0.5}Se_{0.5}/WSe_2$-based photodetectors. (g) The statistical data of photocurrent and dark current, confirming a relatively stable properties of these devices.

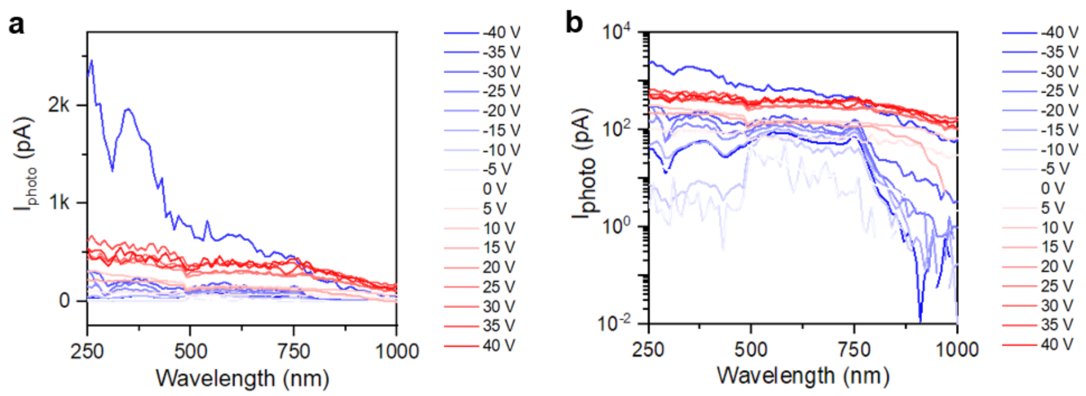

Figure S10. Wavelength-dependent photoresponses based on the gate-tunable 2D-vdWH $GaTe_{0.5}Se_{0.5}/WSe_2$. (a) Spectral response of the 2D-vdWH $GaTe_{0.5}Se_{0.5}/WSe_2$-based photodetector at different V_{gs} in linear coordinates. (b) Spectral response of the 2D-vdWH $GaTe_{0.5}Se_{0.5}/WSe_2$-based photodetector at different V_{gs} in exponential coordinates. The spectral response curves are tested at 5 V intervals in the range of -40 to 40 V. The applied voltage is 2 V and the power density of the illumination is 30 mW/cm^2.

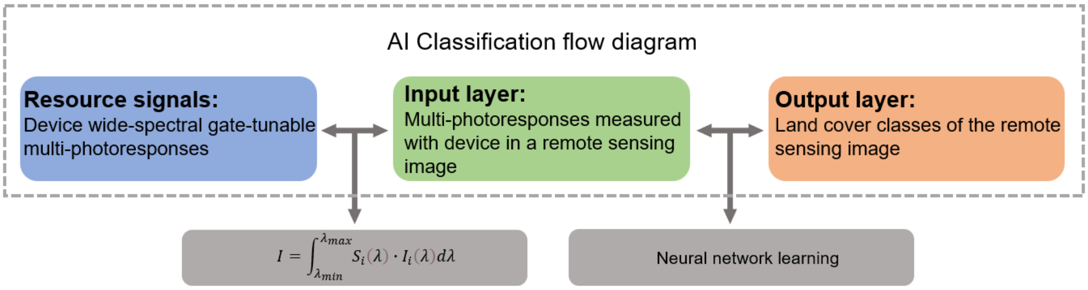

Figure S11. Summarized AI classification flow diagram. Based on the combination of the wide-spectral photoresponses measured under varying gate voltages of the 2D-vdWH $GaTe_{0.5}Se_{0.5}/WSe_2$ and the reflectance spectrum of the remote sensing image, the land cover classes of the remote sensing image can be classified via deep learning algorithms.

Table S1: The optoelectrical performance of $GaTe_{0.5}Se_{0.5}$-based photodetector under the illumination in the spectrum of 254-1064 nm.

Wavelength (nm)	Power density (mW/cm^2)	Responsivity (mA/W)	Detectivity ($*10^9$ Jones)	EQE （%）
254	20	14.27	0.26	7.07
266	40	13.23	0.24	6.07
360	40	35.24	0.63	1.16
450	40	80.15	1.44	22.07
532	40	111.52	2.01	26.07
638	40	135.87	2.45	26.31
808	40	156.22	2.81	23.90
1064	40	106.00	1.91	13.17

Table S2: The optoelectrical performance of 2D-vdWH $GaTe_{0.5}Se_{0.5}/WSe_2$-based photodetector under the illumination in the spectrum of 254-1000 nm.

Wavelength (nm)	Power density (mW/cm^2)	Responsivity (mA/W)	Detectivity ($*10^9$ Jones)	EQE（%）
254	30	20.21	1.20	9.87
360	30	30.15	1.83	10.39
450	30	19.17	1.13	5.28
520	30	42.73	2.56	10.19
638	30	35.42	2.10	6.88
810	30	2.38	0.17	0.36
1000	30	1.62	0.02	0.19

Table S3: Land cover classes images of 6 prevalent hyperspectral remote sensing datasets predicted by neural networks training with the hyperspectral data (250-1000 nm), gate-tunable multi-photoresponses, and non-tunable single photoresponse.

	Hyperspectral data (250-1000 nm)	Multi-photoresponses	Single photoresponse
Samson	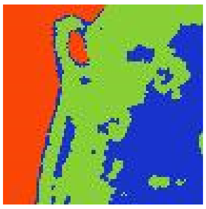	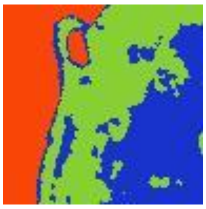	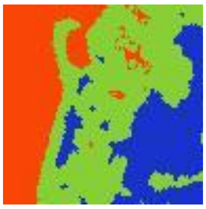
Jasper ridge	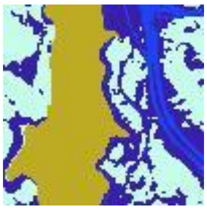	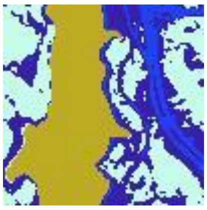	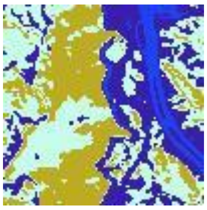
Pavia Center scene	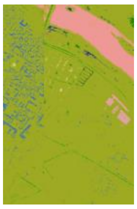	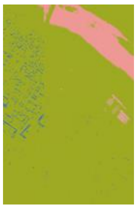	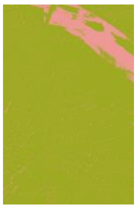
Salinas scene	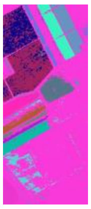	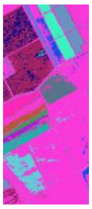	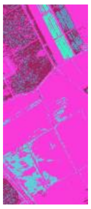
Salinas-A scene	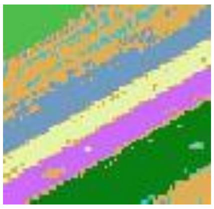	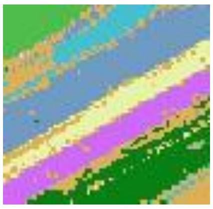	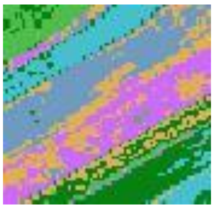
Urban	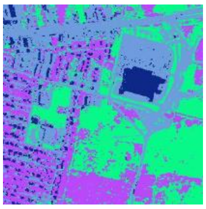	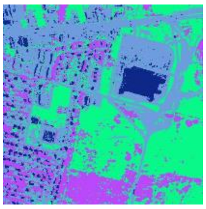	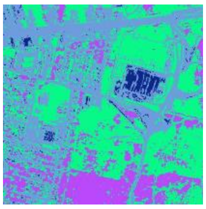
